# Auditory exostosis in Australian warm water surfers: a cross-sectional study

**DOI:** 10.1186/s13102-021-00281-5

**Published:** 2021-05-15

**Authors:** Vini Simas, Wayne Hing, Evelyne Rathbone, Rodney Pope, Mike Climstein

**Affiliations:** 1 Water Based Research Unit, Bond Institute of Health and Sport, Faculty of Health Sciences and Medicine, Bond University, Robina, 2 Promethean Way, Gold Coast, QLD 4226 Australia; 2 Department of Physiotherapy, Faculty of Health Sciences and Medicine, Bond University, Gold Coast, QLD Australia; 3 Faculty of Health Sciences and Medicine, Bond University, Gold Coast, QLD Australia; 4 School of Community Health, Charles Sturt University, Albury, NSW Australia; 5 School of Health and Human Sciences, Southern Cross University, Gold Coast, QLD Australia; 6 Physical Activity, Lifestyle, Ageing and Wellbeing, Faculty Research Group, Faculty of Health Sciences, The University of Sydney, Lidcombe, NSW Australia

**Keywords:** Surfer’s ear, Otology, Sports and exercise medicine

## Abstract

**Background:**

Surfing is a popular sport in Australia, accounting for nearly 10% of the population. External auditory exostosis (EAE), also referred to as surfer’s ear, is recognized as a potentially serious complication of surfing. Cold water (water temperature below 19 °C) is a commonly cited risk factor, with prevalence of EAE in cold water surfers ranging from 61 to 80%.

However, there is a paucity of studies reporting the prevalence of EAE in surfers exposed to water temperatures above 19 °C. With mean water temperature ranging from 19 °C to 28 °C, the Gold Coast region of Australia provides the ideal environment to assess the main goal of this study: to assess the prevalence and severity of EAE in warm water surfers.

**Methods:**

Eligible participants were surfers living and surfing on the Gold Coast (Queensland, Australia). Currently active surfers over 18 years of age, surfing year-round, with a minimum of five consecutive years of surfing experience were recruited to participate. Included individuals were asked to complete a questionnaire and underwent bilateral otoscopy.

**Results:**

A total of 85 surfers were included, with mean age 52.1 years (standard deviation [SD] ±12.6 years) and mean surfing experience of 35.5 years (SD ±14.7 years). Nearly two-thirds of participants (65.9%) had regular otological symptoms, most commonly water trapping (66%), hearing loss (48.2%), and cerumen impaction (35.7%). Less than one-fifth of the surfers (17.7%) reported regular use of protective equipment for EAE. The overall prevalence of exostosis was 71.8%, with most of the individuals having bilateral lesions (59%) and a mild grade (grade 1, 47.5%). There was insufficient evidence for any significant associations between the main outcomes (presence and severity of EAE) and factors related to age, surfing experience, winter exposure, surfing ability, symptoms, and use of protective equipment.

**Conclusion:**

To the best of our knowledge, this is the first study assessing EAE in surfers exposed to warm waters (above 19 °C). The prevalence of 71.8% highlights the high prevalence of the condition in the surfing population, regardless of water temperature. Future research should focus on ways to prevent EAE.

## Key points

- This study examines the prevalence and severity of external auditory exostosis (EAE) in warm water surfers, a relatively unexplored topic in the literature.
- The overall prevalence of EAE in warm water surfers of the Gold Coast region of Australia found in the present study was 71.8%.
- Nearly 60% of the participants had bilateral lesions.
- Recurrence rate of the condition after surgical removal was 50%.
- Future research should focus on effective preventive methods for EAE.

## Background

Surfing is a popular sport in Australia, with the estimated number of participants over 2.5 million, accounting for nearly 10% of the nation’s population [1]. Exostosis of the external auditory canal (EAC), also referred to as surfer’s ear, is recognized as a potentially serious complication of surfing [2, 3]. Commonly multiple and found bilaterally, external auditory exostosis (EAE) is an irreversible benign condition. Exostosis can be associated with a variety of clinical features, including an intermittent blocked feeling of the EAC, especially after water exposure, recurrent cerumen blockage, frequent ear infections, pain in the EAC, and hearing deterioration due to the obstructive nature of the condition [3].

The pathophysiology and prevention of EAE remain unclear. The consistent use of protective equipment, such as earplugs and hoods, has been proposed to prevent its occurrence and is advised [4, 5]; however, the efficacy of these preventative measures remains to be established. Surgery is the only treatment, and it is usually reserved for patients with severe and symptomatic cases; however, the surgical procedure does not prevent recurrence [6, 7].

It is well acknowledged that there is a positive association between the amount of time spent surfing and the presence and severity of EAE, with risk increasing after only five sessions per month and significantly increasing after 5 years surfing [5, 8]. Regarding the relationship between EAE and water temperature, cold water (water temperature below 19 °C) is a commonly cited risk factor, with prevalence of EAE in cold water surfers ranging from 61 to 80% [9–17]. Additionally, anthropological data indicate that regions located more than 30^0^ north or south of the equator line, where the annual average water temperature is below 19 °C, have a high prevalence of EAE [9]. Nevertheless, there is a paucity of studies reporting the prevalence of EAE in surfers exposed to water temperatures above 19 °C.

Located at 28^0^ south of the equator, the Gold Coast region of Australia is world-famous for its surf breaks, with mean water temperature ranging from 19 to 22 °C in August (winter), through to 26 to 28 °C in February (summer) [18]. It thus provides the ideal environment in which to address the main goal of this study: to assess the prevalence and severity of EAE in warm water surfers.

## Methods

### Study design

This research used a cross-sectional observational design, aiming to investigate the prevalence and severity of auditory exostosis in Australian warm water surfers. The study was approved by the Bond University Human Research Ethics Committee (BUHREC 15221).

### Participants

Surfers were recruited through advertising in a local paper and from local boardrider clubs in the Gold Coast (GC) area (City of Gold Coast, Queensland, Australia). Additional support for recruitment was obtained from surfing magazines, websites and local surf shops in the GC area. Participants or the public were not involved in the design, or conduct, or reporting, or dissemination of our research.

#### Eligibility criteria

Only individuals living and surfing in the GC region were considered to be included. Currently active surfers over 18 years of age, surfing all year round, with a minimum of 5 consecutive years of surfing experience, surfing at least five sessions per month, were invited to take part in the research. Surfers were excluded from the study if they had a history of exposure to cold water (mean temperature below 19 °C) for more than 3 consecutive weeks, if they participated for more than 3 consecutive weeks in winter sports activities (e.g., skiing, snowboarding), or if they have lived in cold regions (located more than 30° north or south of the equator) for more than 5 consecutive years in their lifetime. Additionally, participants were excluded if both the right and left EAC were occluded by cerumen.

### Procedures

As described in our previous studies [19, 20], all participants who successfully passed the initial screening were invited to participate in this study. The research took place at the Water Based Research Unit (WBRU), Bond Institute of Health and Sport, Bond University, Gold Coast (Queensland, Australia). An explanatory statement and consent form were given to all participants upon arrival at the WBRU. Prior to providing written informed consent, all potential participants were given the opportunity to ask any questions about the research and about the testing procedure [19, 20]. The explanatory statement illustrated the exam to be conducted, and contained a simple overview of the research project and its purpose [19, 20]. The informed consent form was signed once participants were satisfied with the information provided.

At the WBRU, participants were asked to complete a questionnaire to collect basic demographic data and to examine their surfing habits and otological history, as described in our previous studies [19, 20]. After completing the questionnaire, and in line with the methodology previously adopted by our research team [19, 20], all participants underwent clinical examination of both ears, via otoscopy, by an experienced Sport and Exercise Physician, using a hand-held, battery-powered digital otoscope (Digital MacroView™, Welch Allyn®, USA), capable of acquiring digital images.

### Predictors and outcome measures

#### Surfing characteristics

Surfers were assessed with regard to surfing specific characteristics, which included: surfing experience in years; average number of sessions per week; average number of hours per session; winter exposure in hours (number of hours per session during winter multiplied by number of sessions during winter and number of years surfing); surfing ability, as measured by the Hutt scale [21]; stance while surfing (i.e., ‘regular’ if left foot forward or ‘goofy’ if right foot forward); and main type of surfboard (short, mini-mal/funboard, or longboard). Additionally, they were asked whether they were involved in any other ocean sport.

#### Otological history

Participants were asked about the presence of otological symptoms (e.g., otalgia, hearing loss), regular use of prevention methods for EAE (e.g., ear plug, hood), and previous history of otitis externa (OE) and EAE.

#### Exostosis

**Fig. 1:**
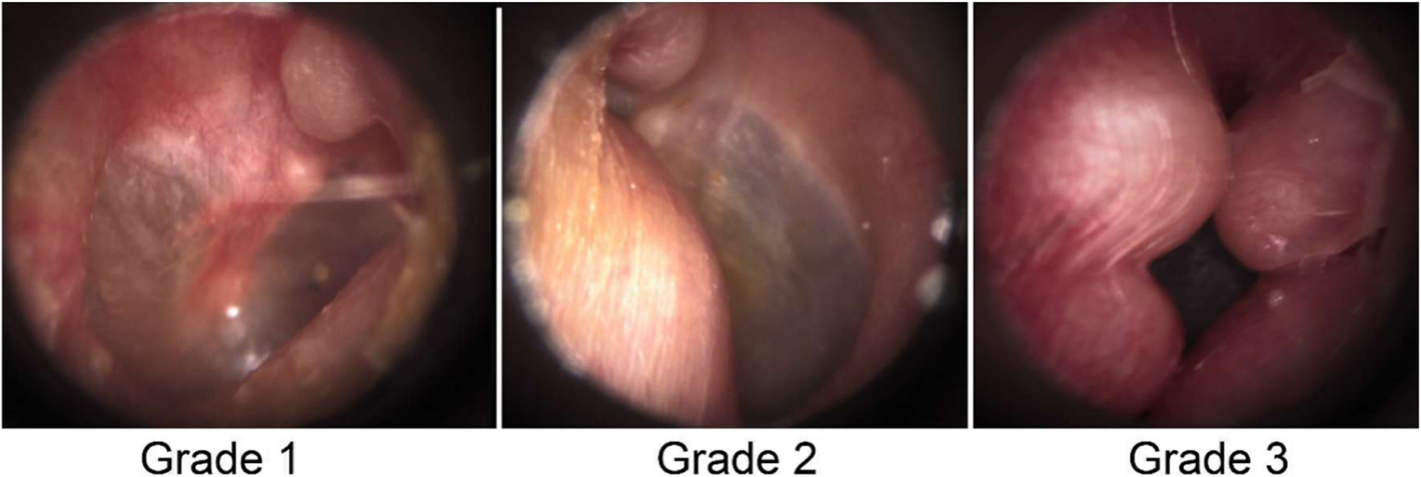
Exostosis severity. Notes: Grade 1: up to 33% of obstruction of the external auditory canal (EAC); Grade 2: between 34 and 66% of obstruction of the EAC; Grade 3: more than 67% of obstruction of the EAC

During otoscopy, images of the EAC were recorded, and all images were assessed to determine the presence of EAE. If present, the degree of obstruction of the EAC was graded on the standard clinical one-to-three scale (Fig. 1; grade 1: up to 33% of obstruction; grade 2: between 34 to 66% of obstruction; grade 3: more than 67% of obstruction), as previously described [16].

#### Reliability

Initially, an intra-rater reliability study was conducted as a single clinician (Specialist) assessed the severity of exostosis in all participants. It is common practice to assess the intra-rater reliability of clinicians [22], particularly when the diagnosis and/or grading is subjective, as is with EAE [23]. A total of 15 EAE images were selected from a pool of approximately 970 images to determine the inter-rater reliability. The reliability between test-retest was 100% by the clinician. We then completed a quantification analyses where we utilized the freely available software *ImageJ*, developed by the National Institutes of Health (Bethesda, MD, USA), which is specifically designed for medical and/or biological analyses with a shown high validity (r = 0.988) and reliability (Cronbach’s alpha = 0.994) [24]. This software is used in a number of medical fields, including computerized tomography analyses, blood vessel diameter analyses, abdominal and skeletal muscle mass, and wound healing [25]. However, to the best of our knowledge, it has not previously been used in determining the severity of exostosis. However, we have recently shown this technique to have a high coefficient of reliability (r = 0.999), and a significant correlation (total area and exostosis area, *p* < 0.01, r = 0.999), and a near perfect positive relationship between repeated measurements [26]. Therefore, we deemed this quantification technique of exostosis appropriate to assess the clinicians analyses of exostoses in our participants. There was 100% agreement between the clinician’s assessment of the severity of exostosis and our quantification method.

### Data analysis

Continuous data were analyzed descriptively to determine means and standard deviations (SD) and tested for normality by assessing skewness, kurtosis, Q-Q plots, and the Kolmogorov-Smirnov test. Categorical outcomes were summarised using frequencies and percentages. A Chi-square test of independence was used to assess associations between the main outcome variables (EAE presence and severity) and categorical outcomes. The level of significance, alpha, was set a priori at 0.05 for all statistical tests. All analyses were performed with SPSS statistical software (Version 25.0 for Windows, SPSS Inc., Chicago, IL, USA, 2017).

## Results

A total of 85 surfers were eligible to take part in our study (eighty-one males, 95.3%), with a mean age of 52.1 years (SD ±12.6). Participants had a mean surfing experience of 35.5 years (SD ±14.7), surfing on average 3.8 times per week (SD ±1.8), and each session having a mean duration of 1.7 h (SD ±0.5). Age, surfing experience, winter exposure, and surfing ability were grouped in categories, and these are shown in Table 1. The majority of the individuals had a regular stance (left foot forward, 78.8%) and used a shortboard (72.9%) as the main type of surfboard. Nearly half of the participants (49.4%) were regularly involved in other ocean sports, most commonly swimming (17.6%) and stand-up paddle boarding (12.9%).

**Table 1:** Demographic characteristics of surfers (*N* = 85)

Characteristics	*n*	Percent
Age		
18 to 30 years	6	7.1%
31 to 50 years	28	32.9%
51 to 75 years	51	60.0%
Surfing experience		
Less than 10 years	6	7.1%
11 to 25 years	20	23.5%
26 to 50 years	46	54.1%
More than 50 years	13	15.3%
Winter exposure		
Less than 1500 h	35	41.2%
1500 to 3000 h	24	28.2%
More than 3000 h	26	30.6%
Surfing ability (Hutt scale [^21^])		
Beginner	2	2.4%
Intermediate	57	67.1%
Advanced	26	30.6%

*Abbreviation*: *n* number of individuals

With regards to otological history, almost two-thirds of the surfers (65.9%) reported having regular symptoms and, of these, 62.5% had two or more symptoms. The most common complaint was water trapping (66%), followed by hearing loss (48.2%) and cerumen impaction (35.7%). Despite the high prevalence of otologic symptomatology, less than one-fifth of the participants (15 individuals) reported regular use of prevention methods. Earplugs (*n* = 5), alcohol-based ear drops (n = 5), and hoods (*n* = 2) were the preventive methods employed by the surfers. Three individuals used a combination of these three methods. Thirty-five surfers (41.2%) reported ear infection in the past, and 20 (23.5%) reported having EAE diagnosed by either their General Practitioner (GP) or a specialist physician, with four of these surfers (20%) having had previous surgical removal of exostosis.

**Fig. 2:**
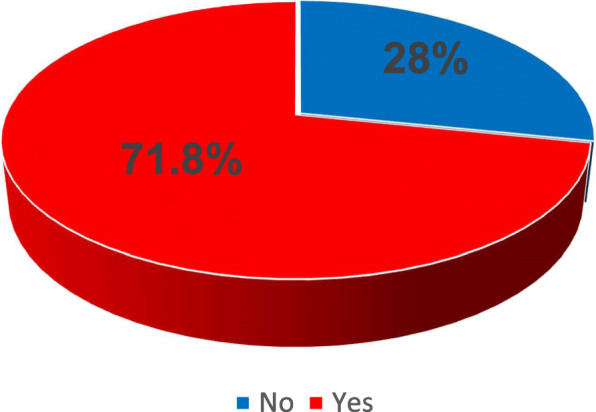
Prevalence of auditory exostosis in 85 surfers

**Fig. 3:**
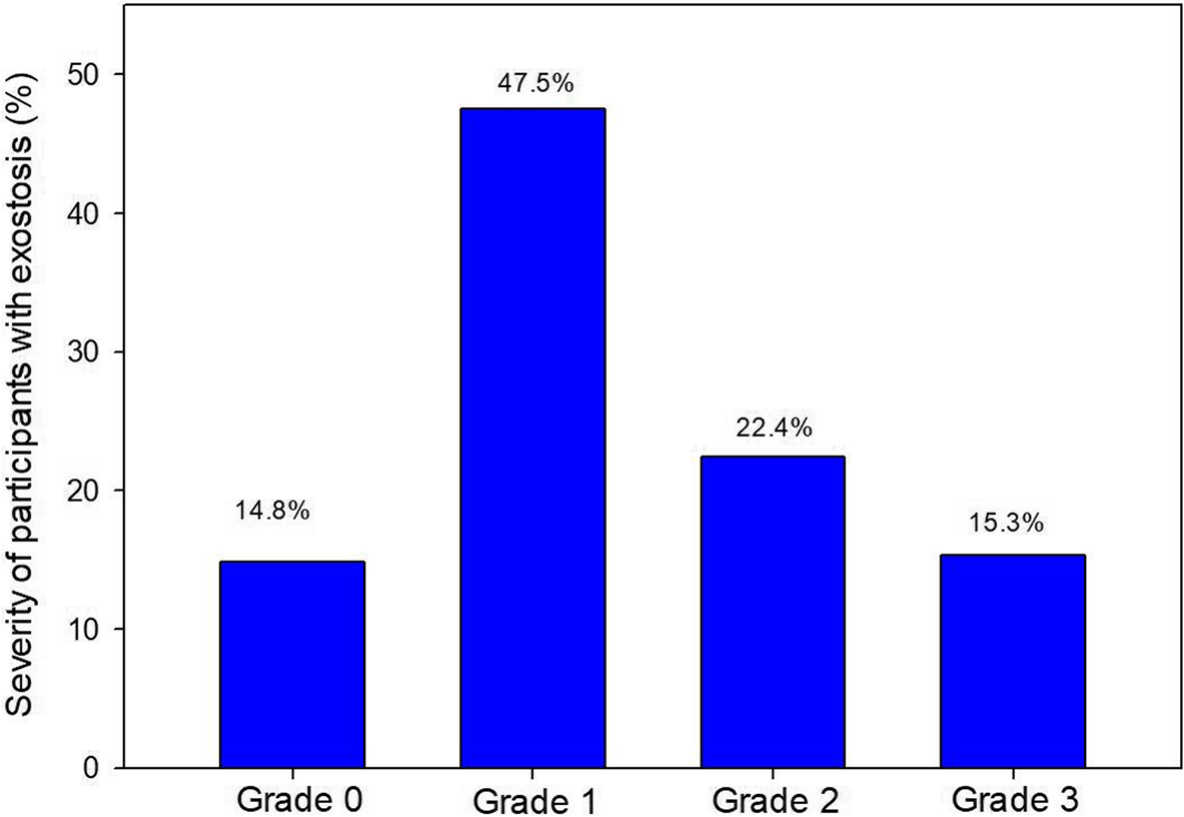
Severity of auditory exostoses in 85 surfers

Some degree of exostosis (grade 1 to 3, inclusive) was present in 61 individuals (71.8%, Fig. 2), of which 36 (59%) had bilateral lesions, with similar prevalence in left and right sides (56.5 and 57.6%, respectively, no significant difference). With regards to severity (Fig. 3), nearly half of the individuals with EAE had only a minor grade (grade 1, 47.5%), 22.4% had grade 2, and only 15.3% were classified as having a severe grade of EAE (grade 3). Of those four individuals who had EAE surgically removed, two (50%) had new lesions on the operated side, and both individuals were classified as having grade 2 (between 34 to 66% of obstruction).

A Chi-square test of independence was used to analyze the association between the presence of EAE and the following predictor variables: age group, surfing experience group, winter exposure group, surfing ability group, participation in other ocean sports, presence of otological symptoms, use of protective equipment, and previous history of OE. There was no correlation between any of the predictor variables and the outcome. Additionally, a Chi-square test of independence was also used to analyze the relationship between the same predictors and the severity of EAE, again with no statistically significant results. However, when analyzing associations between predictor variables and potential cofounding factors, there was a statistically significant association between the presence of hearing loss and age (χ2(1) = 3.901, *p* = 0.048).

## Discussion

### Main findings

The primary goal of the present research was to determine the prevalence of EAE in warm water surfers. To the best of our knowledge, this is the first study to assess the presence of EAE in surfers exclusively exposed to water temperatures above 19 °C. The overall prevalence of the condition in our study was 71.8%. Considering that approximately 10% of the Australian population regularly surf [1], this condition potentially affects nearly 45,000 individuals on the Gold Coast region of Australia.

The current study found that otological symptoms are common in the surfing population, as nearly two-thirds of the participants reported having regular symptoms; however, hearing loss, a common symptom associated with EAE, due to the obstructive characteristic of the condition, was significantly associated with age. Furthermore, we did not find a statistically significant association between symptoms and either the presence or severity of EAE. Also, these outcomes were not associated with the most commonly cited risk factors, namely surfing experience and winter exposure, or with the use of prevention methods.

### Relation to previous studies

This prevalence is comparable to what has been previously reported for cold water surfers, with results ranging from 61 to 80% [9–17]. Moreover, this prevalence is in line with a previous study conducted by Hurst et al. [15] in Victoria, Australia, where the authors reported a prevalence of 76%. Interestingly, a second study conducted in Australia, via an online survey, identified less than 4% of the surfers (3.5%) reported having EAE [27]. A likely explanation for the difference between self-reported and assessed prevalence is the potential lack of awareness of surfers about EAE. It is important to highlight that our study had the highest mean age (52.1 years ±12.6) and the greatest number of years of surfing experience (35.5 years ±14.7), when compared to previous studies. Nonetheless, our main finding highlights that EAE is highly prevalent in surfers, regardless of water temperature. Additionally, it further supports the concept that surfing exposure is potentially the most important predictor of EAE prevalence.

Nearly 60 % of our volunteers had bilateral lesions, consistent with what has been previously reported in the literature. Some studies have suggested that the ear (right or left) more exposed to the prevailing wind would have a higher prevalence of EAE and more severe lesions [10, 15, 28]. The predominant coastal wind on the Gold Coast region is south-southeast (SSE) [29]; however, there was no evidence of a statistical difference in prevalence and severity of EAE between the right and left ears of participants. With regards to severity, the majority of the volunteers in our study (47.5%) only had a mild grade EAE (grade 1), a result that is consistent with previously reported findings. Four individuals (6.6% of those with EAE) who had undergone EAE surgery, had a recurrence rate of 50%. Although surgery is the only treatment for the condition, recurrence is common, even in individuals who stop participation in ocean sports [6, 7].

### Limitations

It is important to note that the findings of this study must be interpreted with caution, mainly due to the study design, but also due to the relatively low number of participants in the study.

## Conclusion

The purpose of the current study was to assess the lifetime prevalence of EAE in warm water surfers. To the best of our knowledge, this is the first study assessing exostosis in surfers surfing exclusively in water temperatures above 19 °C. Our findings revealed a prevalence of 71.8%, demonstrating that exostoses of the EAC are highly prevalent in warm water surfers, just as they are in cold water surfers. Furthermore, the results support the impression that surfing experience is potentially the most important predictor of prevalence. Health practitioners, especially General Practitioners and medical specialists, should be aware of this EAE prevalence in their surfing (and aquatic) patients and approach individuals susceptible to the condition, regardless of water temperature, in order to provide preventive recommendations for this population. Future research should focus on effective preventive methods for EAE.

## Data Availability

All data generated or analysed during this study are included in this published article.

## Abbreviations

EAC: External auditory canal
EAE: External auditory exostosis
BUHREC: Bond University Human Research Ethics Committee
GC: Gold Coast
WBRU: Water Based Research Unit
OE: Otitis externa
SD: Standard deviation
N: Number of individuals

## References

[1] Stark A. Surfing Australia: annual report*.* www.surfingaustralia.com2013. Accessed 15 July 2019.

[2] Zoltan TB, Taylor KS, Achar SA (2005). Health issues for surfers. Am Fam Physician.

[3] Simas V, Furness J, Hing W, Pope R, Walsh J, Climstein M (2016). Ear discomfort in a competitive surfer. Aust Fam Physician.

[4] American Academy of Family Physicians. Information from your family doctor. safe surfing. Am Fam Physician 2005;71:2319–20. 15999869

[5] Alexander V, Lau A, Beaumont E, et al. The effects of surfing behaviour on the development of external auditory canal exostosis. Eur Arch Otorhinolaryngol. 2015;272:1643–9. 10.1007/s00405-014-2950-5 24619201

[6] Hurst W (2001). A review of 64 operations for removal of exostoses of the external ear canal. Aust J Otolaryngol.

[7] Timofeev I, Notkina N, Smith IM (2004). Exostoses of the external auditory canal: a long-term follow-up study of surgical treatment. Clin Otolaryngol Allied Sci.

[8] Deleyiannis FW, Cockcroft BD, Pinczower EF (1996). Exostoses of the external auditory canal in Oregon surfers. Am J Otolaryngol.

[9] Kennedy GE (1986). The relationship between auditory exostoses and cold water: a latitudinal analysis. Am J Phys Anthropol.

[10] Umeda Y, Nakajima M, Yoshioka H (1989). Surfer's ear in Japan. Laryngoscope..

[11] Chaplin JM, Stewart IA (1998). The prevalence of exostoses in the external auditory meatus of surfers. Clin Otolaryngol Allied Sci.

[12] Wong BJ, Cervantes W, Doyle KJ (1999). Prevalence of external auditory canal exostoses in surfers. Arch Otolaryngol Head Neck Surg.

[13] Kroon DF, Lawson ML, Derkay CS, Hoffmann K, McCook J (2002). Surfer's ear: external auditory exostoses are more prevalent in cold water surfers. Otolaryngol--Head Neck Surg.

[14] Altuna Mariezkurrena X, Gómez Suárez J, Luqui Albisua I, Vea Orte JC, Algaba GJ (2004). Prevalence of exostoses surfers of the basque coast. Acta Otorrinolaringol Esp.

[15] Hurst W, Bailey M, Hurst B (2004). Prevalence of external auditory canal exostoses in Australian surfboard riders. J Laryngol Otol.

[16] Nakanishi H, Tono T, Kawano H (2011). Incidence of external auditory canal exostoses in competitive surfers in Japan. Otolaryngol Head Neck Surg.

[17] Attlmayr B, Smith IM (2015). Prevalence of 'surfer's ear' in Cornish surfers. J Laryngol Otol.

[18] The Spit Water Temperature (Sea) and Wetsuit Guide (QLD - Gold Coast, Australia). Surf-forecast.com. http://www.surf-forecast.com/breaks/The-Spit/seatemp. Published 2019. Accessed.

[19] Simas V, Hing W, Furness J, Walsh J, Climstein M. The prevalence and severity of external auditory Exostosis in young to Quadragenarian-aged warm-water surfers: a preliminary study. Sports (Basel). 2020;8(2):17. 10.3390/sports8020017. 10.3390/sports8020017 PMC7077213 32033062

[20] Simas V, Hing W, Pope R, Climstein M (2020). Australian surfers’ awareness of ‘surfer’s ear’. BMJ Open Sport Exerc Med.

[21] Hutt JA, Black KP, Mead ST. Classification of surf breaks in relation to surfing skill. J Coastal Res. 2001;1:66–81. Retrieved July 15, 2019. http://www.jstor.org/stable/25736206.

[22] Osório FL, Loureiro SR, Hallak JEC, Machado-de-Sousa JP, Ushirohira JM, Baes CVW (2019). Clinical validity and intrarater and test-retest reliability of the structured clinical interview for DSM-5 - clinician version (SCID-5-CV). Psychiatry Clin Neurosci.

[23] Lee KC, Bamford A, Gardiner F, Agovino A, ter Horst B, Bishop J (2019). Investigating the intra- and inter-rater reliability of a panel of subjective and objective burn scar measurement tools. Burns..

[24] Lee JH, You J (2016). Movement measurement validity and reliability of the ImageJ program for kinematic analysis. J Mechan Med Biol.

[25] Schindelin J, Rueden CT, Hiner MC, Eliceiri KW (2015). The ImageJ ecosystem: an open platform for biomedical image analysis. Mol Reprod Dev.

[26] Climstein M, Simas V, DeBeliso M, Walsh J. A novel method for the determination of Exostosis severity in the external Auditory Canal. Authorea. January 05, 2021. 10.22541/au.160984548.84730740/v1. 10.1111/coa.13824 34142441

[27] Furness JW, Hing W, Abbott A, Walsh J, Climstein M. Retrospective analysis of acute injuries in recreational and competitive surfers: injury location, type, and mechanism. Int J Aquat Res Educ. 2014;8(3):6. 10.25035/ijare.08.03.06.

[28] King JF, Kinney AC, Iacobellis SF (2010). Laterality of exostosis in surfers due to evaporative cooling effect. Otol Neurotol.

[29] Wind and weather statistic Gold Coast Seaway. Windfinder.com. https://www.windfinder.com/windstatistics/gold_coast_seaway. Published 2019. Accessed.

